# Inverse Association of Plasma Vanadium Concentrations with Gestational Diabetes Mellitus

**DOI:** 10.3390/nu14071415

**Published:** 2022-03-29

**Authors:** Xiaoqin Li, Yalun Zhu, Jiawei Yin, Ben Li, Peiyun Li, Benfeng Cao, Qiang Wang, Jian Xu, Liegang Liu

**Affiliations:** 1 Hubei Key Laboratory of Food Nutrition and Safety, Department of Nutrition and Food Hygiene, School of Public Health, Tongji Medical College, Huazhong University of Science and Technology, 13 Hangkong Road, Wuhan 430030, China; 2019501004@hust.edu.cn (X.L.); 15926237664@163.com (Y.Z.); 2020509033@hust.edu.cn (J.Y.); liben1219@163.com (B.L.); caobenfeng@126.com (B.C.); d201981402@hust.edu.cn (Q.W.); 2 Ministry of Education Key Lab of Environment and Health, School of Public Health, Tongji Medical College, Huazhong University of Science and Technology, 13 Hangkong Road, Wuhan 430030, China; 3 Department of Clinical Nutrition, Wuhan Children’s Hospital (Wuhan Maternal and Child Healthcare Hospital), Tongji Medical College, Huazhong University of Science and Technology, Wuhan 430030, China; lipeiyunhuster@126.com; 4 Shenzhen Center for Chronic Disease Control, No. 2021, Buxin Rd., Shenzhen 518020, China

**Keywords:** vanadium, gestational diabetes mellitus, case-control study

## Abstract

Vanadium compounds were identified to be beneficial for the control of glucose homeostasis. We aimed to explore the association of plasma vanadium (V) with gestational diabetes mellitus (GDM). We performed a case-control study including 252 newly diagnosed GDM cases and 252 controls matched by age, parity, and gestational age. Fasting blood samples were collected from each participant at GDM screening (≥24 weeks of gestation). The plasma concentrations of V were determined utilizing inductively coupled plasma mass spectrometry. Plasma V levels were significantly lower in the GDM group than those in the control group (*p* < 0.001). The adjusted OR (95% CI) of GDM comparing the highest V tertile with the lowest tertile was 0.35 (0.20–0.61). According to the cubic spline model, the relation between plasma V and odds of GDM was potentially nonlinear (*p* < 0.001). Moreover, plasma V was negatively correlated with 1-h post-glucose load, 2-h post-glucose load, and lipid metabolism indices (all *p* < 0.05). The present study indicates an inverse association of plasma V with GDM. Further prospective cohort studies are required to validate our results.

## 1. Introduction

Gestational diabetes mellitus (GDM) refers to any degree of glucose intolerance with onset or first recognition in pregnancy [1]. GDM has serious negative consequences on both mothers and children. During pregnancy, hyperglycemia possibly leads to a series of adverse perinatal outcomes, for example, high caesarean section rate, pre-eclampsia, preterm birth, and neonatal metabolic disturbances [2]. After parturition, those with GDM are more likely to develop GDM or type 2 diabetes mellitus (T2DM) [3]. Meanwhile, their offspring also have an increased risk of diabetes and obesity [4]. Thus, it is imperative to prevent GDM and identifying modifiable factors associated with GDM is of great significance.

Vanadium (V) is a trace element with an essential role in carbohydrates and lipids metabolism and modulations of activities of key enzymes involved in the phosphorylation and dephosphorylation of proteins, kinases, and phosphatases for normal human function [5]. For the general population, the major source of V is food, including mushrooms, shellfish, dill seed, parsley, black pepper, etc. [6]. V deficiency leads to disturbances in thyroid function and the metabolism of glucose and lipid [7]. It is commonly accepted that V compounds exhibit insulin-mimetic properties. In 1985, Heyliger et al. described an anti-diabetic effect of V in streptozotocin-induced diabetic rats [8]. Since then, a large amount of evidence demonstrated beneficial actions of the supplementation of V compounds in various diabetic animals or human patients [9]. Additionally, plasma V concentrations were reported to be negatively associated with newly diagnosed T2DM in a case-control study [10]. However, studies on the potential association of V exposure with GDM are limited and yield inconsistent results [11,12].

Accordingly, we performed a case-control study among the Chinese population to assess whether the plasma levels of V were associated with GDM as well as its metabolic risk factors.

## 2. Materials and Methods

### 2.1. Study Population

In the current hospital-based case-control study, 252 newly diagnosed GDM patients and 252 matched controls were enrolled from Tongji Hospital (Wuhan, China) between August 2012 and April 2015. We included the subjects according to the following criteria: age ≥ 20 years, gestational age ≥ 24 weeks, singleton pregnancy. Participants with a history of GDM, diabetes, or systemic diseases, and those who received medication for regulating glucose metabolism, were excluded.

GDM was diagnosed according to the American Diabetes Association criteria [13]. After overnight fasting, all subjects underwent a 75 g oral glucose tolerance test (OGTT) in the morning. Plasma glucose was measured at fasting, 1 h, and 2 h after the glucose load. GDM was defined if someone had one or more abnormal value: fasting plasma glucose (FPG) ≥ 5.1 mmol/L (92 mg/dL), 1-h plasma glucose ≥ 10.0 mmol/L (180 mg/dL), or 2-h plasma glucose ≥ 8.5 mmol/L (153 mg/dL). Controls were randomly selected from participants with normal glucose levels and 1:1 matched to GDM cases by age (±2 years), gestational age at blood drawing (±2 weeks), and the same parity.

Personal information on sociodemographic characteristics, including age, parity, gestational age, alcohol drinking, smoking habits, and family history of diabetes, were collected through a structured questionnaire by trained investigators. Pre-pregnancy body mass index (BMI) was calculated as weight divided by the square of height (kg/m^2^). Fasting blood was drawn at the time of GDM screening. Then, the plasma was separated and stored at −80 °C for subsequent analysis.

This study was approved by the Ethics Committee of Tongji Medical College (approval No. 2021S044), and all the participants signed informed consents at enrollment. This study has also been registered at www.clinicaltrials.gov (accessed on 10 February 2022) (NCT05267457).

### 2.2. Laboratory Measurements

Plasma total cholesterol (TC), triglycerides (TG), high-density lipoprotein cholesterol (HDL-C), low-density lipoprotein cholesterol (LDL-C), and FPG were analyzed using commercial assay kits (Biosino Bio-Technology and Science Inc., Beijing, China). Fasting plasma insulin (FPI) was measured using enzyme-linked immunosorbent assay kits (Mercodia Company, Uppsala, Sweden). The homeostasis model of assessment-insulin resistance (HOMA-IR) score was computed according to the following formulas: HOMA-IR = FPG (mmol/L) × FPI (μU/mL)/22.5.

The concentrations of plasma V were determined by inductively coupled plasma mass spectrometry (Agilent 7700 Series ICP-MS, Agilent Technologies, Santa Clara, CA, USA) [14]. We set 0.02 μg/L, the concentration of the lowest standard solution, as the limit of quantification for measurement. For quality assurance, the certified reference material ClinChek No. 8883 and No. 8884 human plasma controls were used (1 out of 20 samples). For No. 8883 and No. 8884, the determined concentrations of V were 1.31 ± 0.09 μg/L (certified: 1.11 ± 0.29 μg/L) and 9.28 ± 0.50 μg/L (certified: 9.85 ± 1.97 μg/L). The inter-assay and intra-assay coefficients of variation were both <5%.

### 2.3. Statistical Analysis

Data were summarized according to cases and controls, as mean ± standard deviation (SD) (parametrically distributed) or median with interquartile ranges (IQRs) (nonparametrically distributed) if continuous, and as number (percentage) if categorical. Comparisons of differences in each pair of continuous variables between groups were assessed using Student’s *t*-test or Mann–Whitney U test. The Chi-square test was adopted to detect differences in categorical variables. Participants were categorized into tertiles defined according to the distribution of V among the control subjects. Conditional logistic regression analysis was utilized to estimate the strength of the association of plasma V with GDM by odds ratios (ORs) and 95% confidence intervals (CIs). The regression model was adjusted for age, gestational age at blood sample collection, pre-pregnancy BMI, family history of diabetes (yes or no), drinking habits (yes or no), and smoking status (yes or no). The median value of each tertile of V was considered as a continuous variable in the logistic regression models to test for a linear trend. A logarithmic transformation was adopted to make the distribution of plasma V follow a normal distribution. The potential nonlinearity of the association of plasma V with odds of GDM was further examined using a restricted cubic spline with three knots at the 25th, 50th, 75th percentiles of ln (plasma V concentrations) used via Stata version 13 (StataCorp). Correlation between plasma V levels and TC, TG, LDL-C, HDL-C, FPG, FPI, HOMA-IR, 1-h post-glucose load, and 2-h post-glucose load were performed using Pearson or Spearman correlation coefficient. Additionally, we also calculated the partial correlation coefficients after adjusting the confounding factors mentioned before.

Statistical analyses were carried out with SPSS software package 24.0 (SPSS Inc., Chicago, IL, USA). A *p* value < 0.05 (two-tailed) was considered statistically significant.

## 3. Results

In Table 1, the basic characteristics of the 504 subjects (252 GDM cases and 252 non-GDM controls) were presented. No significant between-group differences were found in age, parity, and gestational age. GDM cases have a higher proportion of family history of diabetes, higher pre-pregnancy BMI, higher levels of FPG, OGTT-1h, OGTT-2h, FPI, HOMA-IR index, TG, and LDL cholesterol than controls without GDM. In addition, compared to controls, GDM cases had a significantly lower concentration of plasma V (*p* < 0.001).

**Figure 1:**
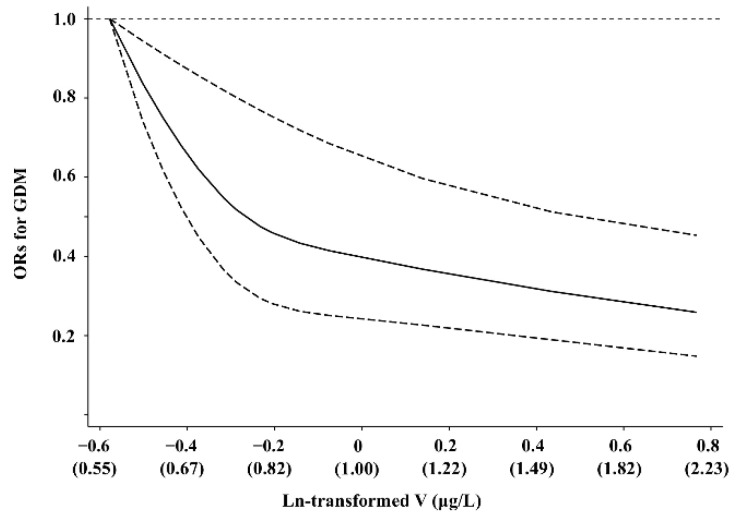
Representation of restricted cubic spline regression for ln-transformed plasma V and GDM. The solid line indicated ORs derived from restricted cubic spline regression, with knots at 25th, 50th, and 75th percentiles of ln-transformed plasma V concentrations and dashed lines indicating 95% CIs. Results were adjusted with age, gestational age at blood sample collection, pre-pregnancy body mass index, family history of diabetes, drinking habits, and smoking status. V, vanadium; GDM, gestational diabetes mellitus.

Higher levels of plasma V were associated with lower odds of GDM. The crude ORs (95% CI) of GDM across increasing tertiles of plasma V levels were 1 (referent), 0.48 (0.30–0.76), 0.40 (0.25–0.65), respectively (Table 2). Moreover, the association remained statistically significant after adjusting for age, gestational age at blood sample collection, pre-pregnancy BMI, family history of diabetes, drinking habits, and smoking status (*p*-trend = 0.002). For each SD increment of ln-transformed plasma V, the risk of GDM decreased 32% (OR: 0.68; 95% CI: 0.56, 0.84). In addition, the potential nonlinearity of the relation between plasma V and odds of GDM was found in the restricted cubic spline model (*p* < 0.001) (Figure 1). The odds of GDM reduced dramatically when the concentration of plasma V was less than 0.82 μg/L, while they declined slightly afterward.

As displayed in Table 3, plasma V was negatively correlated with FPG, OGTT-1h, OGTT-2h, FPI, HOMA-IR, and LDL cholesterol (all *p* < 0.05). After adjusting aforementioned confounding factors, V concentrations still maintained significantly inverse associations with OGTT-1h (*r* = −0.10, *p* = 0.040), OGTT-2h (*r* = −0.09, *p* = 0.043), TC (*r* = −0.09, *p* = 0.046), TG (*r* = −0.10, *p* = 0.030), and LDL cholesterol (*r* = −0.14, *p* = 0.002).

## 4. Discussion

In the present case-control study, we found an inverse association of plasma V concentrations with odds of GDM, independent of potential confounding risk factors of GDM. Moreover, plasma V was negatively correlated with OGTT-1h, OGTT-2h, TC, TG, and LDL cholesterol.

Previous studies on the relation between the levels of V and GDM are limited and yield inconsistent results. One case-control study reported that V exposure, reflected by meconium V concentrations, was negatively associated with GDM risk in 137 GDM cases and 197 controls [15], which was similar to our findings. In a prospective cohort study, Zhang et al. found that serum V had no statistically significant association with GDM, but inversely correlated with OGTT-1h (*β* = −0.09, *p* = 0.014) [12], which was partly consistent with our results. The participants in the current study had lower blood V concentrations (median (IQR): 0.73 (0.63–0.89) μg/L in GDM cases and 0.80 (0.70–1.11) μg/L in controls) than those in Zhang’s study (median (IQR): 1.96 (0.44–2.46) μg/L), which might contribute to the discrepancies in findings. In addition, an opposite result was observed in another Chinese cohort study, which reported that V exposure in early pregnancy was positively associated with GDM risk [11]. However, this study used urinary V levels as the exposure biomarker and we used blood concentrations. Due to the absence of evidence on the relationship between blood V and urine V, it is hard to compare these two results directly. Moreover, our findings were consistent with the results of studies focusing on T2DM among non-pregnant populations. A case-control study in the Chinese population, including 223 T2DM cases and 302 controls, reported that serum V was inversely associated with T2DM risk [16]. Similarly, another study including 802 T2DM cases and 796 controls indicated a negative relationship of plasma V concentrations with the odds of T2DM [10]. Furthermore, previous studies have shown that V intervention yielded beneficial effects on glucose metabolism in animal models with impaired glucose regulation [17,18] and patients with non-insulin-dependent diabetes mellitus [19]. Accordingly, our study provided convincing support to the assumption that V had a beneficial role in the control of glucose homeostasis.

Though an inverse relationship of plasma V with GDM was indicated in our study, the exact biological mechanisms have not been clarified. One of the mechanisms is likely to be related to the protein kinase pathway [20]. The phosphate analog vanadium compounds could bind to the active site of protein tyrosine phosphatase (PTP) to inhibit PTP activity and activate phosphatidylinositol 3-kinase signaling through the enhancement of tyrosine phosphorylation of insulin receptors, thereby leading to the translocation of glucose transporter 4 to the cell membrane [21]. Vanadium-induced phosphatidylinositol 3-kinase activation pathway has been found to play a critical role in mediating vanadyl sulfate- and sodium orthovanadate-induced stimulation of glucose uptake [22], glycogen synthesis [23], and glucose transporter translocation [24] in varieties of cells. On the other hand, vanadium compounds may also exert the insulin-sensitizing effect through the protein kinase B-dependent transduction pathway by increasing adiponectin level [25].

Besides, since dyslipidaemia has been identified as one of the major risk factors of GDM [26], the preventive effect of V on GDM may be partly through improving lipid metabolism. According to previous studies, three weeks of vanadyl sulfate oral treatment improved hepatic and skeletal muscle insulin sensitivity in diabetic patients, and, meanwhile, suppressed the level of plasma free fatty acids and lipid oxidation [27,28]. Treatment with vanadyl sulfate on streptozocin-induced diabetic rats reversed abnormal levels of serum TC, TG, LDL-C, HDL-C, and phospholipid [29]. The hypoglycaemic effect is possibly mediated by inducing autophagy via activating the liver kinase B-1 (LKB1) and adenosine mono-phosphate-activated protein kinase (AMPK) signaling pathway to reduce hepatic lipid accumulation according to intervention studies in vivo and in vitro [30,31,32]. The LKB1/AMPK signal pathway has been shown to be indispensable in lipid metabolic regulation [30,33].

Our study displayed several strengths. To exclude the artificial interference after GDM diagnosis, we confined all the GDM patients to the newly diagnosed patients, because treatments such as lifestyle changes may distort the association. Furthermore, all the GDM cases were matched with controls by age, parity, and gestational age to minimize the potential confounding data derived from these factors. However, several potential limitations still exist. First, as a case-control study, it could not allow us to infer causality relationships. That is to say, it is not yet possible to decide whether low levels of V were causing GDM, or whether the disease was leading to lower V concentrations, or whether there is even a self-amplifying cycle between them. Therefore, further prospective studies need to be conducted to confirm our findings. Second, all the participants were enrolled from a certain city in China, which makes this study population relatively homogenous in ethnic background and V exposure and enhances the internal validity of our findings. However, it restricts the generalizability of these results to other populations. Third, although there were various confounding factors, we still lack the information on other residual confounding variables that may have an impact on the association we examined.

## 5. Conclusions

In conclusion, our results suggest that higher plasma V concentrations are associated with lower odds of newly diagnosed GDM in a Chinese population. Further prospective cohort studies are required to validate our results.

## Figures and Tables

**Table 1:** Characteristics of GDM and control groups.

Variables	GDM (*n* = 252)	Controls (*n* = 252)	*p*
Age (years)	30.05 ± 3.76	29.56 ± 3.74	0.161
Parity, *n* (%)			1.000
1	203 (80.56)	203 (80.56)	
≥2	49 (19.44)	49 (19.44)	
Gestational age at blood sample collection (wk)	28.49 ± 2.85	28.45 ± 3.09	0.903
Pre-pregnancy BMI (kg/m^2^)	22.22 ± 3.18	20.89 ± 2.80	<0.001
Family history of diabetes, *n* (%)	65 (25.79)	35 (13.89)	0.001
Alcohol drinking, *n* (%)	12 (4.76)	12 (4.76)	1.000
Smoking, *n* (%)	4 (1.59)	6 (2.38)	0.523
FPG (mmol/L)	5.24 (5.06–5.45)	4.70 (4.57–4.90)	<0.001
OGTT-1h (mmol/L)	9.60 (8.52–10.94)	7.55 (6.50–8.49)	<0.001
OGTT-2h (mmol/L)	8.62 (7.51–9.47)	6.96 (6.17–7.72)	<0.001
FPI (μU/mL)	10.36 (7.71–14.16)	8.27 (5.96–10.50)	<0.001
HOMA-IR	2.44 (1.75–3.33)	1.74 (1.25–2.28)	<0.001
TC (mmol/L)	5.49 (4.78–6.28)	5.36 4.71–6.05)	0.185
TG (mmol/L)	2.59 (2.00–3.18)	2.27 (1.74–3.04)	0.002
LDL cholesterol (mmol/L)	3.24 (2.51–3.99)	3.02 (2.36–3.72)	0.047
HDL cholesterol (mmol/L)	1.34 (1.17–1.56)	1.38 (1.13–1.64)	0.512
V (μg/L)	0.73 (0.63–0.89)	0.80 (0.70–1.11)	<0.001

Abbreviations: GDM, gestational diabetes mellitus; BMI, body mass index; FPG, fasting plasma glucose; OGTT-1h, 1-h post-glucose load; OGTT-2h, 2-h post-glucose load; FPI, fasting plasma insulin; HOMA-IR, homeostasis model of assessment-insulin resistance; TC, total cholesterol; TG, triglycerides; LDL, low-density lipoprotein; HDL, high-density lipoprotein; V, vanadium. Data were presented as *n* (%) for categorical data, means ± SDs for parametrically distributed data, or median (IQRs) for nonparametrically distributed data.

**Table 2:** Association of plasma V concentrations with GDM.

	Tertiles of Plasma V Concentration			*p*-Trend	Per SD Increment of ln-Transformed Plasma V
	Tertile 1 (<0.68 μg/L)	Tertile 2 (0.68–0.97 μg/L)	Tertile 3 (≥0.97 μg/L)		
No. of cases/controls	129/84	65/84	58/84		
Crude model	1	0.48 (0.30–0.76)	0.40 (0.25–0.65)	0.001	0.72 (0.61–0.86)
Model 1 ^a^	1	0.51 (0.32–0.80)	0.41 (0.25–0.67)	0.002	0.70 (0.58–0.85)
Model 2 ^b^	1	0.46 (0.28–0.78)	0.35 (0.20–0.61)	0.002	0.68 (0.56–0.84)

Abbreviations: V, vanadium; GDM, gestational diabetes mellitus; SD, standard deviation. ^a^ Model 1: adjusted for age (years) and gestational age at blood collection (weeks). ^b^ Model 2: adjusted for model 1 plus pre-pregnancy body mass index (kg/m^2^), family history of diabetes (yes/no), drinking habits (yes/no), and smoking (yes/no).

**Table 3:** Correlation coefficients between V and metabolic characteristics.

Variables	Unadjusted		Adjusted ^a^	
	*r*	*p*	*r*	*p*
FPG (mmol/L)	−0.15	0.001	−0.05	0.294
OGTT-1h (mmol/L)	−0.15	0.001	−0.10	0.040
OGTT-2h (mmol/L)	−0.17	0.001	−0.09	0.043
FPI (μU/mL)	−0.09	0.048	−0.04	0.396
HOMA-IR	−0.09	0.036	−0.03	0.482
TC (mmol/L)	−0.05	0.291	−0.09	0.046
TG (mmol/L)	−0.05	0.246	−0.10	0.030
LDL cholesterol (mmol/L)	−0.11	0.016	−0.14	0.002
HDL cholesterol (mmol/L)	0.06	0.195	0.09	0.051

Abbreviations: V, vanadium; FPG, fasting plasma glucose; OGTT-1h, 1-h post-glucose load; OGTT-2h, 2-h post-glucose load; FPI, fasting plasma insulin, HOMA-IR, homeostasis model of assessment-insulin resistance; TC, total cholesterol; TG, triglycerides; LDL, low-density lipoprotein; HDL, high-density lipoprotein. ^a^ Partial correlation, adjusted for age (years), pre-pregnancy body mass index (kg/m^2^), gestational age at blood sample collection (weeks), family history of diabetes (yes/no), drinking habits (yes/no), and smoking (yes/no).

## Data Availability

The data presented in this study are available on reasonable request from the corresponding author.
